# A generalized heterogeneous federated model for identifying patients with postoperative progression of early-stage non-small cell lung cancer

**DOI:** 10.1038/s41598-025-30565-6

**Published:** 2025-12-01

**Authors:** Jun Xu, Bao Feng, Xiaojuan Chen, Senliang Lu, Fei Wu, Zhaole Yu, Kunwei Li, Qiong Li, Qinggeng Jin, Wansheng Long, Huan Lin, Yehang Chen, Xiangmeng Chen

**Affiliations:** 1 School of Computer and Electronic Information, Guangxi University, Nanning, China; 2 Laboratory of Intelligent Detection and Information Processing, Guilin University of Aerospace Technology, Guilin, China; 3 Jiangmen Key Laboratory of Artificial Intelligence in Medical Image Computation and Application, Jiangmen Central Hospital, Jiangmen, Guangdong China; 4 Department of Radiology, Jiangmen Central Hospital, Jiangmen, 529030 Guangdong China; 5 IFlytek Technology Co., Ltd., Artificial Intelligence Cloud Service Platform R&D Building, No. 666 Wangjiang West Road, High-Tech Zone, Free Trade Pilot Zone, Hefei, Anhui China; 6 Department of Radiology, The Fifth Affiliated Hospital of Sun Yat-Sen University, Zhuhai, China; 7 Department of Radiology, Sun Yat-Sen University Cancer Center, Guangzhou, China; 8 School of Electrical Engineering, Guangxi University, Nanning, China; 9 Department of Radiology, Guangdong General Hospital, Guangzhou, China

**Keywords:** Early-stage non-small cell lung cancer, CT image classification, Robust feature transfer, Federated learning, Cancer, Computational biology and bioinformatics, Mathematics and computing, Oncology

## Abstract

In the postoperative management of early-stage non-small cell lung cancer (NSCLC), accurate identification of patients at high risk of progression is essential for developing personalized follow-up schedules and adjuvant treatment strategies. However, in multi-center settings, model and data heterogeneity limit the flexibility and generalizability of traditional federated learning. To address this, we propose a heterogeneous federated learning model (HFLM) that enables centers to adopt different model architectures while using a robust feature transfer strategy to alleviate the impact of heterogeneous data. Using CT images from 892 early-stage NSCLC patients across four medical institutions, HFLM achieved AUCs of 0.863 (95% CI, 0.8072–0.9192), 0.837 (95% CI, 0.7204–0.9530), 0.846 (95% CI, 0.7349–0.9564), and 0.847 (95% CI, 0.6971–0.9963). Cross-validation and stratified analyses further confirm its strong generalization and stability across centers.

**Supplementary Information:**

The online version contains supplementary material available at 10.1038/s41598-025-30565-6.

## Introduction

Lung cancer is the leading cause of cancer-related deaths^1^. It can be classified into two major types: non-small cell lung cancer (NSCLC) and small cell lung cancer (SCLC), with NSCLC accounting for about 85% of all cases^2^. Despite significant advancements in early diagnosis, personalized treatment, and targeted therapies in recent years, the overall prognosis for lung cancer remains poor, and survival rates are still low^3^. Currently, the most important prognostic factor for predicting lung cancer recurrence and survival is the TNM staging system^4,5^. However, due to its limitations, some patients within the same stage group experience long-term non-progression after surgery, while others progress rapidly with recurrence and metastasis^6^. Therefore, building on existing methods, it is particularly important to explore approaches that can further improve the accuracy of risk stratification in order to better guide personalized treatment and improve patient outcomes.

With the development of computer technology, federated learning algorithms have been widely applied in research areas such as lung cancer^7,8^. Federated learning is a distributed learning approach that trains a shared global model by aggregating locally updated model parameters without directly accessing the data held by clients. Based on federated averaging (FedAvg)^9^, Li et al. proposed FedProx^10^, which introduces a regularization term to the objective function of each client to mitigate the parameter divergence between local and global models. Model-contrastive federated learning (MOON)^11^ enhances the consistency between global and local model parameters by employing contrastive learning to construct a loss function based on the cosine similarity of features between the global and local models.

Typically, federated learning achieves a global model through averaging aggregation, which requires client devices to have sufficient computational resources to train deep models and adequate communication resources to exchange model parameters with the server^9–11^. However, the computational resources and communication capabilities of each client vary greatly and often dynamically change according to their current load, which can make those with minimal resources a bottleneck for federated learning. To address this issue, clients should train personalized local models of varying architectures according to their available resources. As a result, when centers with different model architectures participate in federated learning, model heterogeneity arises. This heterogeneity causes inconsistencies in hierarchical structures and parameter dimensions, making it impossible to directly transfer or adapt global model parameters for local personalized training. These structural differences also prevent parameter-by-parameter matching or weighted averaging during global model aggregation, further complicating the process. Therefore, we need to consider how to build local models of different architectures while reducing the impact of multi-center data heterogeneity on the convergence and performance of the global model; thereby effectively handling the model heterogeneity issue to enhance the generality and practicality of federated learning.

Driven by these real-world issues, we propose a universal heterogeneous federated learning model architecture that allows each center to train different model architectures based on its own needs. By introducing a robust feature transfer strategy^12^, we designed a transfer loss function for local model training and global model aggregation, effectively addressing the model heterogeneity issue. At the same time, this strategy reduced the introduction of heterogeneous features during feature transfer, further improving the overall performance of the model. Experimental results demonstrated that this universal architecture achieves significant improvements in both local and global model prediction performance, validating its superiority in heterogeneous federated learning scenarios. Additionally, we evaluated the generalization ability and stability of the HFLM model through cross-validation.

## Materials & methods

### Patients

This study utilized retrospective data collection, including clinical and imaging data from patients with solid NSCLC who underwent curative surgical resection and were pathologically confirmed between January 2014 and September 2019 at four medical centers. The research was approved by the Ethics Committee of Jiangmen Central Hospital (Center A, *n* = 455), Guangdong Provincial People’s Hospital (Center B, *n* = 188), the Fifth Affiliated Hospital of Sun Yat-sen University (Center C, *n* = 141), and the Cancer Center of Sun Yat-sen University (Center D, *n* = 108). Details of the CT scanners at each center are provided in Supplementary Material Table S1. The study was conducted in accordance with the 1964 Declaration of Helsinki and its subsequent amendments or similar ethical standards. This study was approved by the Ethics Committee of Jiangmen Central Hospital. The ethics review approval number: Ethics Review of Jiangmen Central Hospital No.[2021]3 A. The Ethics Committee of Jiangmen Central Hospital waived both the requirement for written informed consent and the necessity for participation consent due to the retrospective nature of the study and the use of anonymized data.

The inclusion criteria were as follows: (1) patients with solid non-small cell lung cancer (NSCLC) who underwent surgical resection and were pathologically confirmed; (2) preoperative chest CT scans performed within one month prior to surgery; (3) CT images retrievable from the Picture Archiving and Communication System (PACS); (4) complete and regular postoperative follow-up records for at least three years; (5) pathological staging of I or II; (6) chest CT slice thickness of ≤ 1.5 mm. The exclusion criteria were as follows: (1) patients with concurrent malignancies; (2) patients with incomplete medical records; (3) patients received neoadjuvant therapy before surgery.

Follow-up protocol: (1) for the first two years post-surgery, repeat chest CT scans every 6 to 12 months. Thereafter, conduct annual chest CT scans; (2) in the event of clinical symptoms, perform additional examinations as necessary; (3) the study endpoint is reached when disease progression is observed. Patients without disease progression will be followed up until they complete three years. After the aforementioned screening process, 892 patients were included in the study. To ensure that the data distributions between the training set and the test set were similar, thereby allowing for more accurate model performance evaluation, an adversarial validation strategy was employed to partition the data from the four hospitals. Patient demographics are provided in Table 1.

**Table 1:** Basic patient information.

Center	Set	Disease type	Age(mean ± std)	Gender		CEA		Smoking history	
				Male	Female	Negative	Positive	Absence	Presence
Center A(455)	Train(276)	NP-ELC(229)	60.46 ± 10.177	120	109	178	51	172	57
		P-ELC(47)	62.77 ± 8.911	34	13	34	13	34	13
	Test(179)	NP-ELC(146)	59.82 ± 10.497	78	68	122	24	108	38
		P-ELC(33)	59.82 ± 11.221	16	17	19	14	24	9
Center B(188)	Train(104)	NP-ELC(85)	60.59 ± 9.810	46	39	69	16	64	21
		P-ELC(19)	66.11 ± 9.474	11	8	10	9	10	9
	Test(84)	NP-ELC(66)	61.23 ± 11.109	39	27	53	13	48	18
		P-ELC(18)	64.33 ± 12.190	13	5	8	10	11	7
Center C(141)	Train(78)	NP-ELC(62)	58.21 ± 10.238	38	24	50	12	36	26
		P-ELC(16)	56.44 ± 12.806	9	7	13	3	10	6
	Test(63)	NP-ELC(54)	58.04 ± 10.259	24	30	42	12	31	23
		P-ELC(9)	56.44 ± 6.616	5	4	6	3	3	6
Center D(108)	Train(60)	NP-ELC(51)	58.98 ± 12.225	27	24	37	14	35	16
		P-ELC(9)	61.44 ± 6.912	5	4	7	2	6	3
	Test(48)	NP-ELC(41)	60.12 ± 9.314	21	20	32	9	27	14
		P-ELC(7)	57.57 ± 5.318	4	3	6	1	6	1

NP-ELC: non-progression early-stage lung cancer, P-ELC: progression early-stage lung cancer, Std standard deviation.

### Definition of early-stage lung cancer progression

The definition of progression in this study primarily follows the opinions of scholars Toba^13^ and Shimada^14^. Progression is defined as follows: (1) during postoperative follow-up surveillance, recurrence or metastasis is determined through a comprehensive assessment using physical examination, imaging techniques, and tumor marker testing; (2) Local recurrence is defined as the appearance of lesions at the surgical margins, within the ipsilateral thoracic cavity, or in the mediastinum; (3) distant metastasis refers to the presence of lesions in the contralateral lung, thoracic cavity, or any organs outside the mediastinum; (4) where clinically feasible, histopathological or cytological examinations should be performed to confirm the diagnosis; (5) suspected recurrent or metastatic lesions that cannot be confirmed by histopathology are clinically diagnosed as recurrence or metastasis if they increase in size during continued follow-up or decrease in size following antitumor therapy^13,14^.

### ROI acquisition

**Fig. 1:**
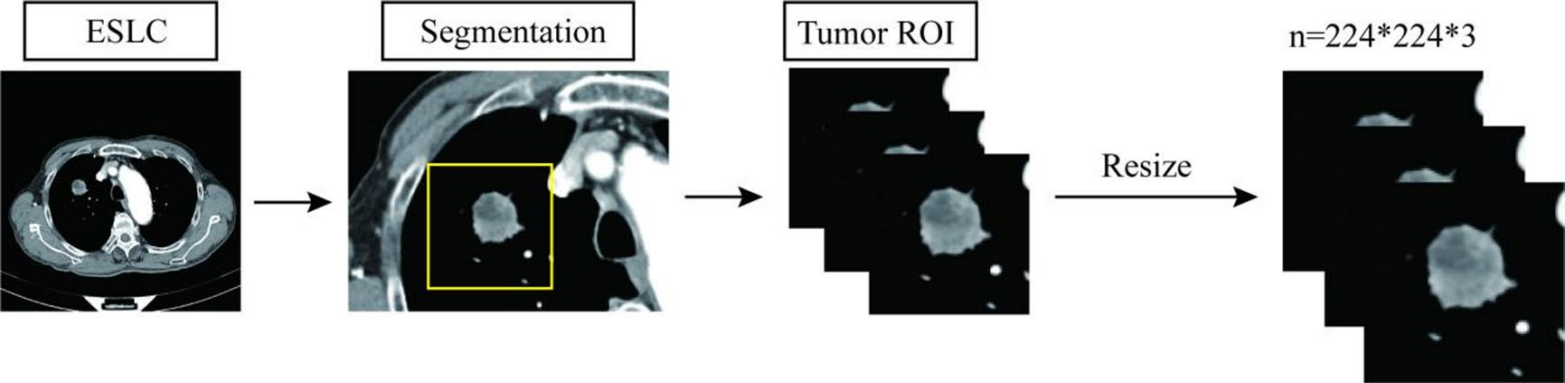
The ROI extraction process. ESLC Early-stage Lung cancer.

In the initial stage of the study, radiologists with over 10 years of experience in thoracic imaging manually segmented consecutive axial images from the venous phase of contrast-enhanced chest CT scans. A manual segmentation algorithm was used to delineate the regions of interest (ROIs), with each ROI covering the entire tumor within a rectangular area. Subsequently, all segmented lesion images were resized and normalized to 224 × 224 × 3 pixels, as illustrated in Fig. 1. This size was chosen because the deep learning models used in this study were pretrained on the large-scale ImageNet dataset, whose standard input size is 224 × 224 × 3. Using this resolution allows direct utilization of pretrained weights, thereby fully leveraging the powerful feature extraction capabilities obtained through transfer learning.

### Construction of the HFLM

**Fig. 2:**
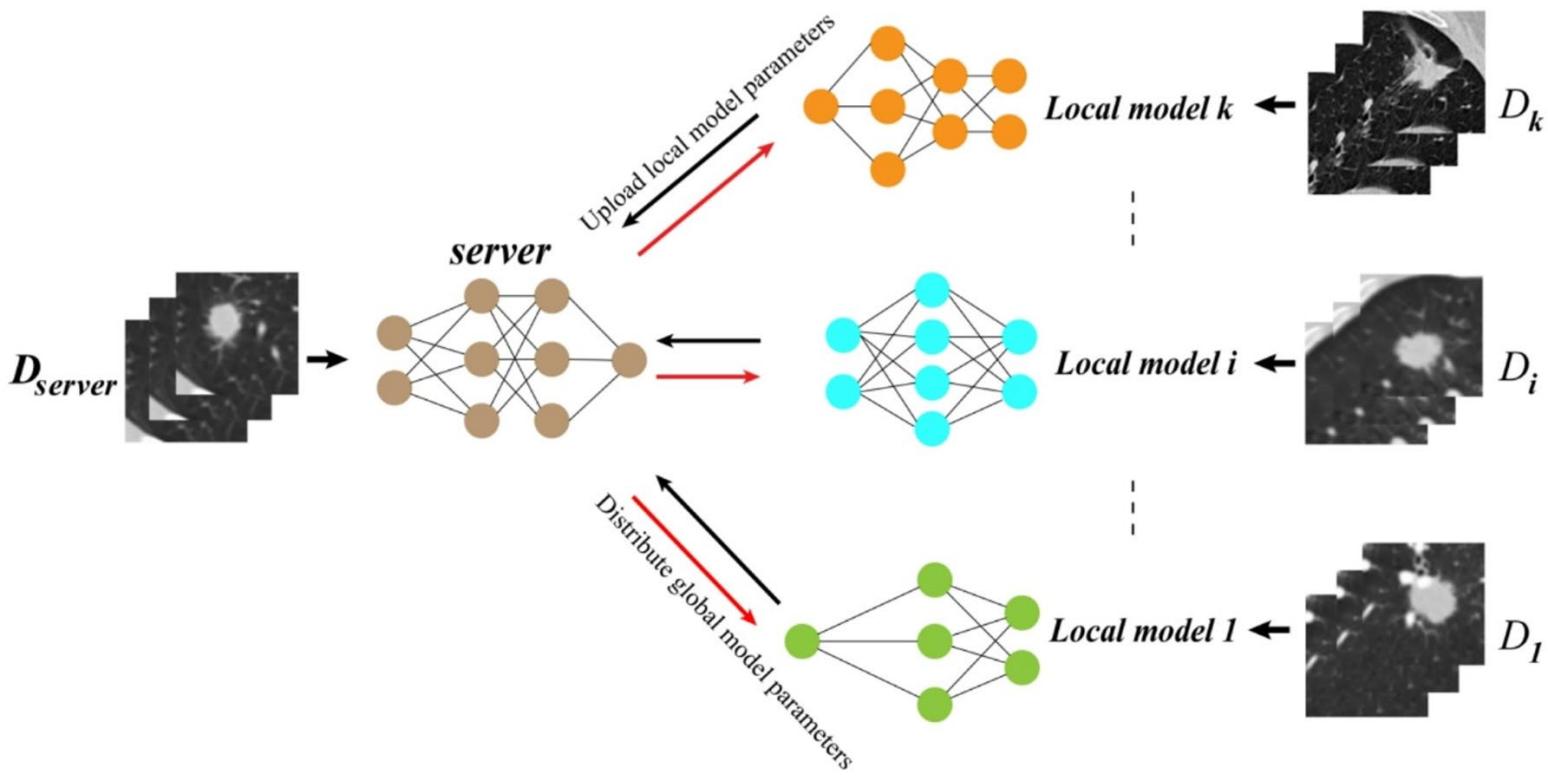
The heterogeneous federated learning algorithm framework diagram.

**Fig. 3:**
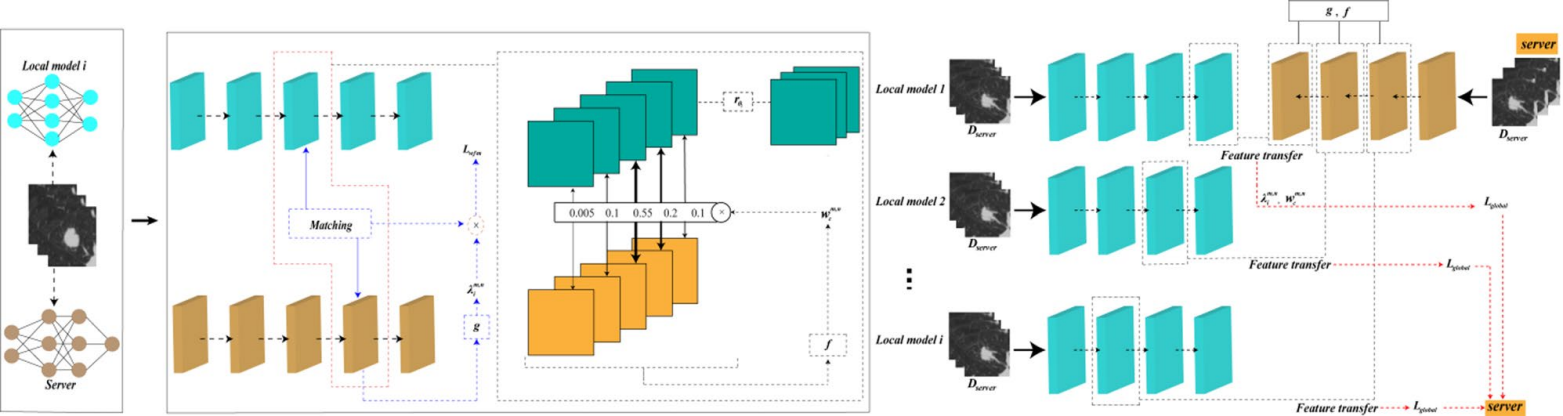
Personalized training of local models and global model aggregation based on robust feature transfer.

To address the issues of data heterogeneity and model heterogeneity in multi-center scenarios, we introduced a strategy of robust feature transfer to build a heterogeneous federated learning model (HFLM). The HFLM framework diagram is shown in Fig. 2, where we assume one center as the server and other centers act as clients. The HFLM consists of two key components: personalized training of local models and aggregation of the global model (Fig. 3). In the personalized training phase of the local models, the server sends global model parameters to each client. However, due to the model heterogeneity issue, local models cannot directly use the global model parameters. Therefore, we construct adaptive heterogeneous model interaction networks for each center based on a robust feature transfer strategy to guide the training of local models and improve their predictive performance.

To achieve this, the robust feature transfer strategy enables each local model to evaluate the relevance and consistency of incoming feature representations from the global model. By assigning dynamic importance weights to different feature channels, the strategy emphasizes informative, task-relevant features while suppressing noisy or incompatible ones. This selective transfer mechanism enhances robustness by aligning transferred features with local data characteristics, reducing interference from structural discrepancies across models. In this way, robust features are more effectively leveraged to improve local model generalization under heterogeneous conditions.

The heterogeneous model interaction networks transfer feature knowledge from the global model according to local data characteristics and construct a transfer loss function based on transfer weights to constrain local model training. Since this feature knowledge is highly relevant to the local data characteristics, it helps reduce the impact of heterogeneous features, thereby improving the model’s predictive performance on local data.

During the global model aggregation phase, the server collects model parameters from each client. However, in the presence of model heterogeneity, it is not possible to assign fixed weights to the parameters of each local model, as in traditional aggregation methods, and directly aggregate the global model parameters. Therefore, we construct a dynamic aggregation network. This network is designed to transfer robust features from local models to the global model. It calculates the feature transfer weights for each local model based on global data characteristics and adaptively updates the transfer weights according to changes in the global model’s cross-entropy. Through iterative training, the network gradually filters out heterogeneous feature information that does not contribute to the global model’s performance, while the robust features that persist in each local model are used to aggregate the global model parameters. In each cycle of information exchange, the server must receive local model parameters from all clients and transmit the updated global model parameters back to the clients. Detailed information about the algorithm is provided in Supplementary S1. The pseudocode for the feature transfer-based model training is provided in Table S2.

After model training was completed, the CNN models trained at each center were used to extract deep features from the local datasets. Specifically, the global model was implemented using the ResNet-18 architecture, and the local models were implemented using the VGG-16 architecture, to simulate a heterogeneous model scenario in federated learning and verify the effectiveness and robustness of the proposed algorithm when different clients use different model structures. To obtain richer feature representations, we did not rely solely on the final output of ResNet-18 but extracted intermediate features from its different network blocks. These feature maps were concatenated to form a 1,536-dimensional global feature representation. For the VGG-16 local models, we adopted the same feature extraction strategy, resulting in a 1,472-dimensional local feature representation. To enhanced prediction performance and reduced computational complexity, the Mann-Whitney U test was used for feature selection, followed by dimensionality reduction with the Maximum Relevance Minimum Redundancy (mRMR) algorithm. Finally, the selected features were used for classification with a Sparse Bayesian Extreme Learning Machine (ELM). Detailed information is provided in Supplementary S2.

### Evaluation and comparison of models

To comprehensively evaluated the performance of HFLM in a multi-center environment, this study conducted comparisons with other models, including a clinical feature-based CM model, traditional federated learning models (FedAvg^9^, FedProx^10^, and Moon^11^, and a combined data training model (CDTM). In this study, the clinical features included age, gender, smoking history and Carcinoembryonic Antigen(CEA) status, and the multivariate logistic regression algorithm was used to construct clinical models for the four data centers(Supplementary S3). To demonstrate the effectiveness of the robust feature transfer strategy in heterogeneous federated learning, we designed a comparison of HFLM with the heterogeneous federated learning algorithm FedProto^15^ and knowledge distillation-based methods(KD).

Additionally, to further validate the generalization ability, stability, and reliability of HFLM, we performed five-fold cross-validation independently within each center, with each center’s dataset randomly partitioned. This approach allows a comprehensive assessment of the impact of variations in data distribution on model performance.

To further investigate the decision basis and interpretability of HFLM in different patient categories, we employed the Gradient-weighted Class Activation Mapping (Grad-CAM) method to visualize the model’s attention regions. Specifically, the trained HFLM model was applied to CT images from each center to extract the activation features of the last convolutional layer. The gradients of the target class were then used to weight these feature maps, producing heatmaps that highlight the key regions contributing to the model’s predictions. Finally, the heatmaps were overlaid onto the original CT images to intuitively demonstrate the differences in model attention between progressive and non-progressive patients.

### Statistical analyses

To thoroughly assess the performance of the compared algorithms, we adopted multiple evaluation indicators, including area under the curve (AUC), specificity, sensitivity, accuracy, positive predictive value (PPV), and negative predictive value (NPV). In addition, integrated discrimination improvement (IDI) and net reclassification index (NRI) were applied to determine whether the HFLM achieved significant improvements in predicting postoperative progression of early-stage lung cancer. The 95% confidence intervals (CIs) for AUCs were calculated using DeLong’s test, which estimates the standard error of the AUC based on the covariance between positive and negative samples; the specific calculation process is provided in Supplementary S4. These measures are widely recognized for evaluating classification models and facilitating statistical comparisons across methods. The receiver operating characteristic (ROC) curves were further plotted to visualize the overall discriminative ability of the models. Robustness analysis indicated that the proposed model was relatively insensitive to variations in local data distribution.

Statistical analyses were performed using two-tailed tests, and the p value < 0.05 was considered statistically significant. Specifically, R software (version 4.4.1) was used for advanced statistical plotting, while IBM SPSS Statistics 26 was applied for basic descriptive statistical analyses.

For deep learning, the following hardware and software configurations were employed: Graphics card: NVIDIA RTX A6000, CUDA version 10.2, GPU memory: 48GB; Deep learning framework: PyTorch (GPU version); Programming language: Python 3.10.13; MATLAB version: 2020b. In this workflow, Python together with CUDA was used as the core modeling tool to implement the federated learning algorithms and accelerate computations via GPU parallelization. MATLAB was utilized for initial preprocessing of medical images and constructing certain classifiers. Together, these tools enabled a complete workflow from data preprocessing and model training to result analysis. The global model and local models were initialized with ResNet18 and VGG16 models pre-trained on ImageNet, respectively. The parameters for building the deep learning model are detailed in Table S3.

## Results

### The HFLM predicts postoperative progression in early-stage lung cancer patients

Compared to the clinical model, the HFLM demonstrated superior diagnostic performance in predicting postoperative outcomes for early-stage lung cancer. Across the four central test sets, its overall accuracy increased by 22.65%.(**Table S4**).

The superior performance of the HFLM over the clinical model was likely attributable to its ability to extract task-specific, highly relevant features, enabling it to more effectively distinguish between CT images of local progression and non-progression in early-stage lung cancer patients. This advantage was further validated by the IDI and NRI, which confirmed that HFLM significantly outperformed the clinical model across data from all centers (Table 2).

**Table 2:** Model evaluation improvement sheet.

Center	IDI		NRI	
	Model2	Model1(Clinical model)	Model2	Model1(Clinical model)
CenterA NP-ELC(146) P-ELC(33)	HFLM	0.2702(*P* = 0.00000)	HFLM	1.1432(*P* = 0.00000)
CenterB NP-ELC(66) P-ELC(18)	HFLM	0.2079(*P* = 0.00462)	HFLM	0.8586(*p* = 0.0.0002)
CenterC NP-ELC(54) P-ELC(9)	HFLM	0.1819(*P* = 0.01026)	HFLM	0.8889(*P* = 0.00779)
CenterD NP-ELC(41) P-ELC(7)	HFLM	0.2002(*P* = 0.01069)	HFLM	0.9895(*P* = 0.00673)

IDI: integrated discrimination improvement. NRI: net reclassification improvement, HFLM: heterogeneous federated learning model. Statistical test: Z-test (two-tailed). P: significance value.

**Fig. 4:**
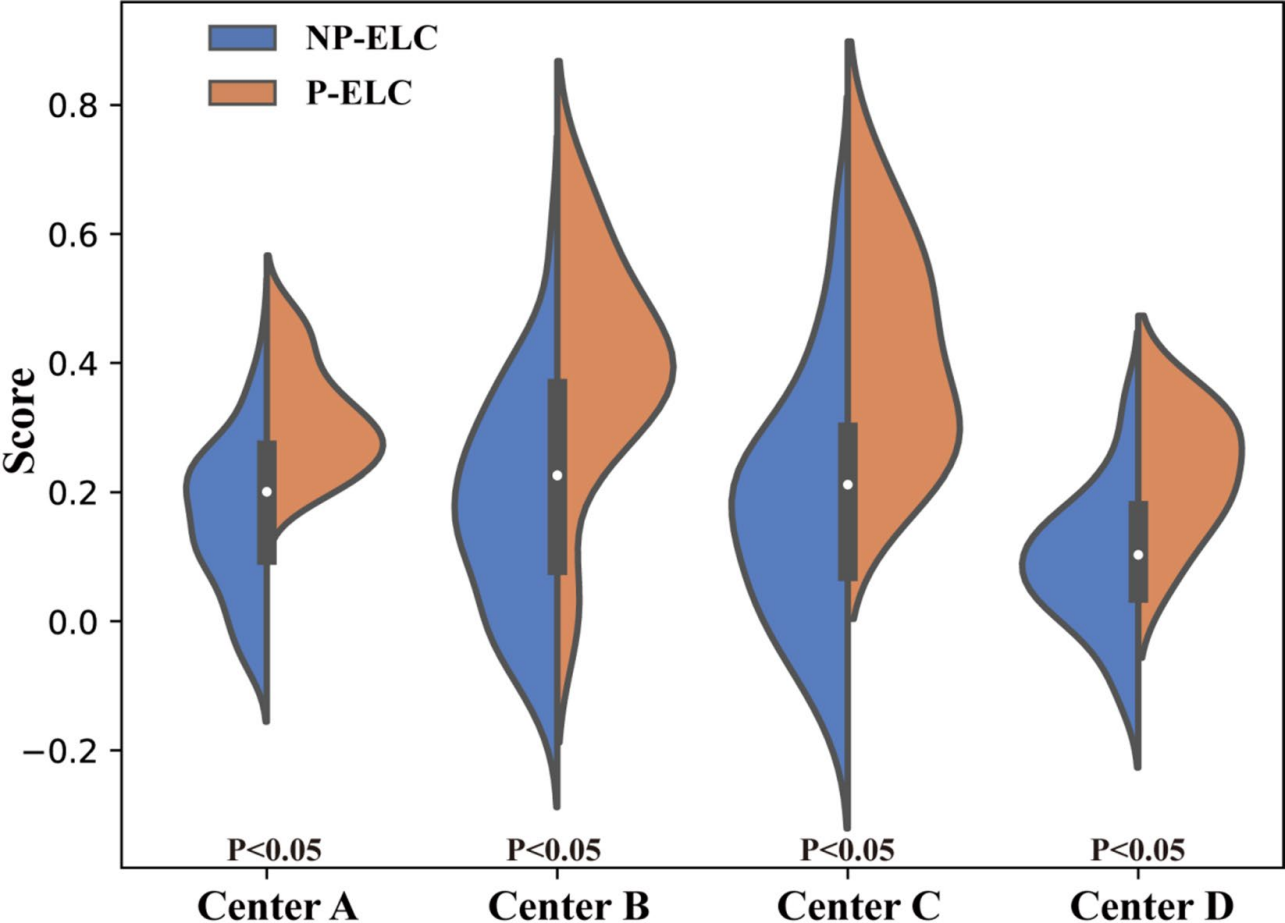
The score charts illustrate the positive and negative images of the four data centers evaluated by the *HFLM*. *HFLM*: heterogeneous federated learning model, *NP-ELC*: non-progression early-stage lung cancer, *P-ELC*: progression early-stage lung cancer.

Figure 4 presents the score distributions of non-progression (NP-ELC) and progression (P-ELC) early-stage lung cancer cases across four data centers, evaluated using the HFLM. Overall, the score distributions of NP-ELC and P-ELC show some differences across the centers, with relatively small overlap between the two categories. This indicates that the model has a reasonable ability to distinguish between the two types of cases.

### The HFLM outperforms other models

**Fig. 5:**
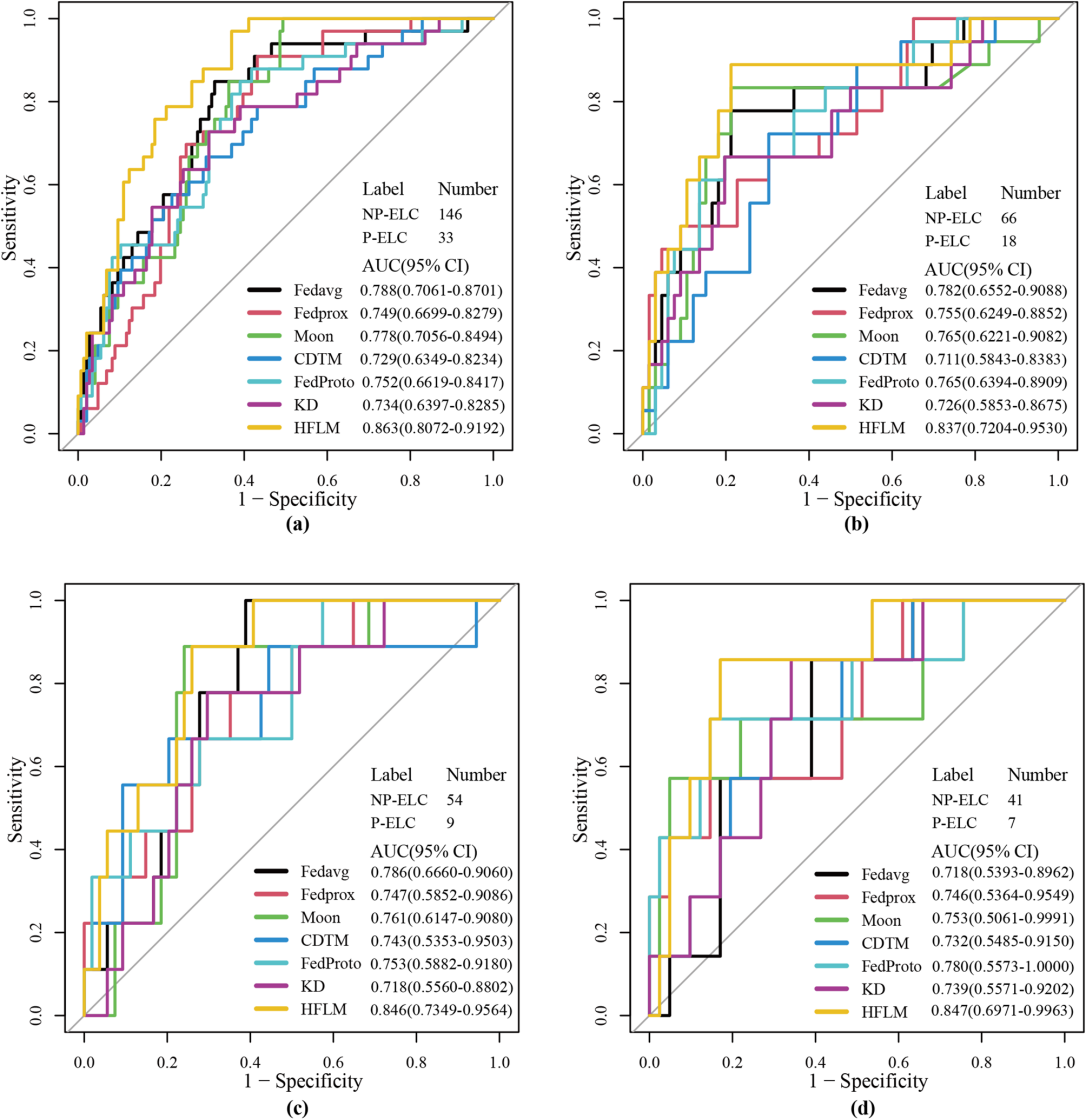
ROC curves of the seven algorithms across the four centers. ROC, receiver operating characteristic.

**Fig. 6:**
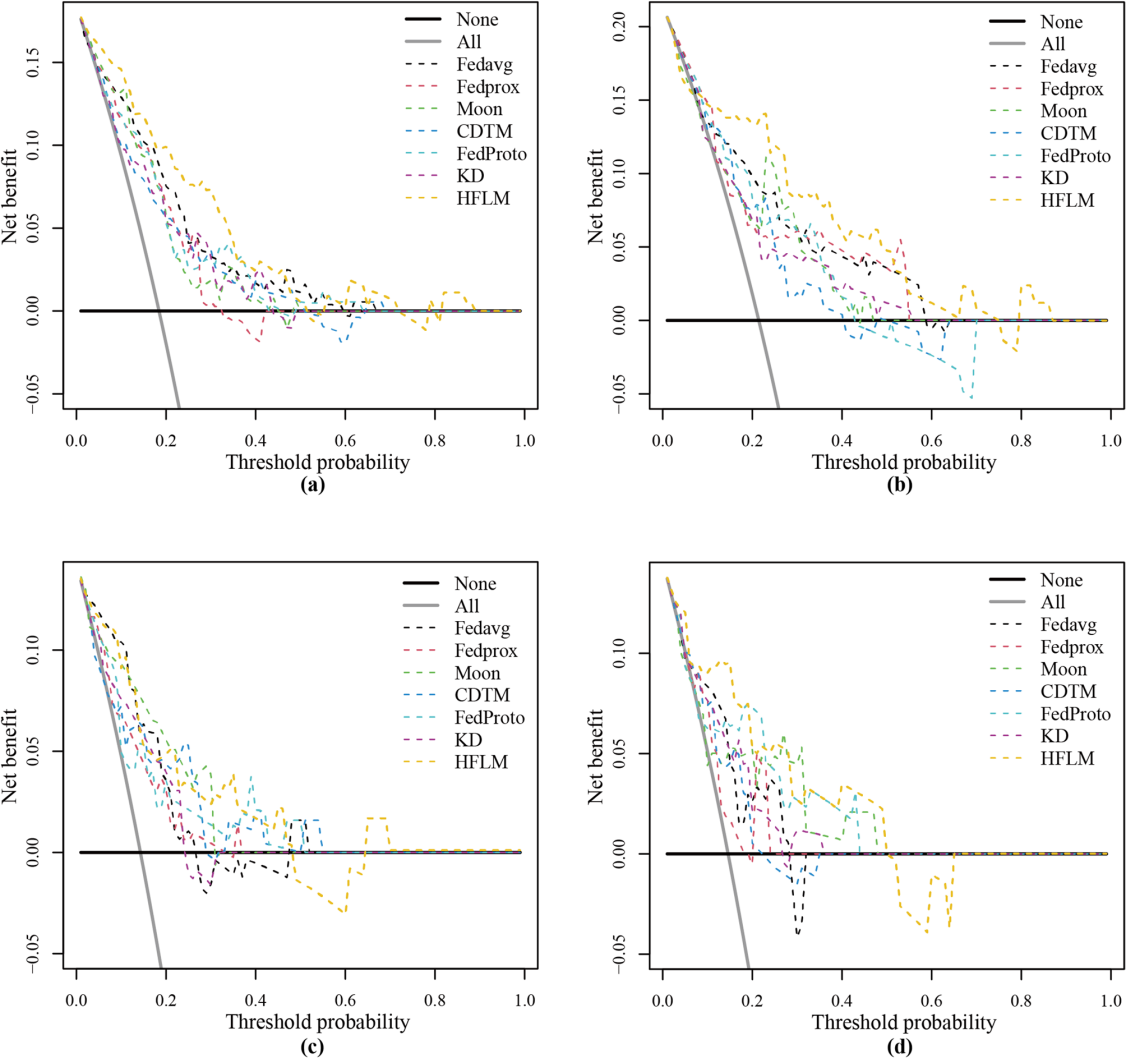
Decision curve analysis of test set of four centers.

To further evaluate the performance of HFLM, we compared it with four federated learning models, including FedAvg^9^, FedProx^10^, Moon^11^, and FedProto^15^, as well as a knowledge distillation-based framework (KD). In addition, a combined data training model (CDTM) was introduced as a reference. Figure 5 presents the ROC curves of all models across the four centers, where HFLM achieved the highest AUC values of 0.863, 0.837, 0.846, and 0.847, respectively. Furthermore, as shown in Fig. 6, the decision curve analysis (DCA) indicates that HFLM provides a higher net clinical benefit across a wide range of threshold probabilities, further demonstrating its potential clinical applicability. The classification threshold was determined based on the predicted probabilities in the training set by selecting the optimal cut-off point, and it was subsequently applied to the test set. Clinically, this threshold indicates that patients with predicted progression probabilities above the cut-off are considered high-risk and may require closer follow-up or adjuvant therapy, whereas those below the cut-off may continue with routine surveillance. Detailed diagnostic performance metrics are shown in Table 3. The confusion matrices for each center are shown in Figure S1.

**Table 3:** Diagnostic performance of the seven models for progression and non-progression classification tasks on early-stage lung cancer datasets from four centers.

Center	Methods	AUC (95% CI)	Sensitivity	Specificity	Accuracy	PPV	NPV
Center A	Fedavg	0.788 (0.7061–0.8701)	0.758 (25/33)	0.692 (101/146)	0.704 (126/179)	0.357 (25/70)	0.927 (101/109)
	Fedprox	0.749 (0.6699–0.8279)	**0.848** (28/33)	0.582 (85/146)	0.631 (113/179)	0.315 (28/89)	0.944 (85/90)
	Moon	0.778 (0.7056–0.8494)	**0.848** (28/33)	0.623 (91/146)	0.665 (119/179)	0.337 (28/83)	0.948 (91/96)
	CDTM	0.729 (0.6349–0.8234)	0.727 (24/33)	0.603 (88/146)	0.626 (112/179)	0.293 (24/82)	0.907 (88/97)
	FedProto	0.752 (0.6619–0.8417)	0.758 (25/33)	0.637 (93/146)	0.659 (118/179)	0.321 (25/78)	0.921 (93/101)
	KD	0.734 (0.6397–0.8285)	0.636 (21/33)	0.705 (103/146)	0.693 (124/179)	0.328 (21/64)	0.896 (103/115)
	HFLM	**0.863** (0.8072–0.9192)	**0.848** (28/33)	**0.712** (104/146)	**0.737** (132/179)	**0.400** (28/70)	**0.954** (104/109)
Center B	Fedavg	0.782 (0.6552–0.9088)	0.833 (15/18)	0.621 (41/66)	0.667 (56/84)	0.375 (15/40)	0.932 (41/44)
	Fedprox	0.755 (0.6249–0.8852)	0.611 (11/18)	**0.742** (49/66)	**0.714** (60/84)	0.393 (11/28)	0.875 (49/56)
	Moon	0.765 (0.6221–0.9082)	0.833 (15/18)	0.606 (40/66)	0.655 (55/84)	0.366 (15/41)	0.930 (40/43)
	CDTM	0.711 (0.5843–0.8383)	0.778 (14/18)	0.530 (35/66)	0.583 (49/84)	0.311 (14/45)	0.897 (35/39)
	FedProto	0.765 (0.6394–0.8909)	0.722 (13/18)	0.636 (42/66)	0.655 (55/84)	0.351 (13/37)	0.894 (42/47)
	KD	0.726 (0.5853–0.8675)	0.778 (14/18)	0.545 (36/66)	0.595 (50/84)	0.318 (14/44)	0.900 (36/40)
	HFLM	**0.837** (0.7204–0.9530)	**0.889** (16/18)	0.667 (44/66)	**0.714** (60/84)	**0.421** (16/38)	**0.957** (44/46)
Center C	Fedavg	0.786 (0.6660–0.9060)	**0.889** (8/9)	0.630 (34/54)	0.667 (42/63)	0.286 (8/28)	0.971 (34/35)
	Fedprox	0.747 (0.5852–0.9086)	0.556 (5/9)	0.741 (40/54)	0.714 (45/63)	0.263 (5/19)	0.909 (40/44)
	Moon	0.761 (0.6147–0.9080)	0.778 (7/9)	0.759 (41/54)	**0.762** (48/63)	0.350 (7/20)	0.954 (41/43)
	CDTM	0.743 (0.5353–0.9503)	0.556 (5/9)	**0.796** (43/54)	**0.762** (48/63)	0.313 (5/16)	0.915 (43/47)
	FedProto	0.753 (0.5882–0.9180)	0.667 (6/9)	0.519 (28/54)	0.540 (34/63)	0.188 (6/32)	0.903 (28/31)
	KD	0.718 (0.5560–0.8802)	0.778 (7/9)	0.648 (35/54)	0.667 (42/63)	0.269 (7/26)	0.946 (35/37)
	HFLM	**0.846** (0.7349–0.9564)	**0.889** (8/9)	0.741 (40/54)	**0.762** (48/63)	**0.364** (8/22)	**0.976** (40/41)
Center D	Fedavg	0.718 (0.5393–0.8962)	0.571 (4/7)	0.829 (34/41)	0.792 (38/48)	0.364 (4/11)	0.919 (34/37)
	Fedprox	0.746 (0.5364–0.9549)	0.286 (2/7)	**0.951** (39/41)	**0.854** (41/48)	**0.500** (2/4)	0.886 (39/44)
	Moon	0.753 (0.5061–0.9991)	**0.714** (5/7)	0.756 (31/41)	0.750 (36/48)	0.333 (5/15)	0.939 (31/33)
	CDTM	0.732 (0.5485–0.9150)	0.286 (2/7)	0.829 (34/41)	0.750 (36/48)	0.222 (2/9)	0.872 (34/39)
	FedProto	0.780 (0.5573−1.0000)	0.571 (4/7)	0.854 (35/41)	0.813 (39/48)	0.400 (4/10)	0.921 (35/38)
	KD	0.739 (0.5571–0.9202)	**0.714** (5/7)	0.707 (29/41)	0.708 (34/48)	0.294 (5/17)	0.935 (29/31)
	HFLM	**0.847** (0.6971–0.9963)	**0.714** (5/7)	0.854 (35/41)	0.833 (40/48)	0.455 (5/11)	**0.946** (35/37)

AUC area under the curve, PPV positive predictive value, NPV negative predictive value.

Significant values are in bold.

For the task of classifying early-stage lung cancer progression and non-progression, as shown in Table 3, Fedavg achieved high sensitivity at Center C, however, the increase in sensitivity typically comes at the cost of decreased specificity. Fedprox demonstrated higher specificity at Center B but had lower sensitivity. Additionally, Fedprox showed higher specificity, accuracy, and PPV at Center D, but its sensitivity was very low. At Center C and Center D, due to the larger discrepancy between positive and negative samples, our method (HFLM) scored slightly lower than FedProx and Moon on certain metrics. In addition, the combined data training model (CDTM), which was trained by merging the training data from all centers, exhibited lower generalization ability across individual centers. This performance decline is likely due to the data heterogeneity among centers, where simply combining data from multiple institutions leads to the model overfitting to dominant data distributions while underperforming on minority distributions. Compared with FedProto, our HFLM achieved better overall balance between sensitivity and specificity across most centers. While FedProto can enhance inter-center feature alignment through prototype representation, its performance was more sensitive to intra-class variance caused by imaging heterogeneity, leading to unstable classification results in minority classes. Furthermore, compared with the knowledge distillation-based framework (KD), which transfers global model knowledge to local clients, HFLM achieved higher stability and accuracy. The KD approach improved convergence speed but exhibited limited adaptability to non-IID data, as the distilled teacher information often failed to capture the local feature distributions.

Nevertheless, our method (HFLM) still achieved superior overall performance. A comprehensive comparison of AUC, sensitivity, specificity, accuracy, PPV and NPV shows that our method (HFLM), through its robust feature transfer strategy, not only effectively addressed model heterogeneity but also reduced the introduction of heterogeneous features, thereby enhancing the model’s generalization capability. Consequently, in multi-center heterogeneous settings, HFLM demonstrated strong performance across all centers without exhibiting poor performance at any specific center.

### The HFLM exhibits strong robustness

**Fig. 7:**
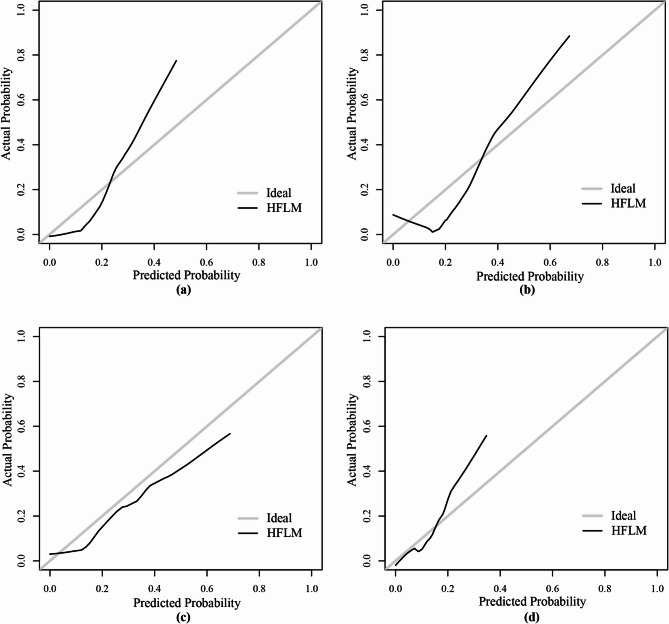
Calibration curves of HFLM across the four centers.

To assess the calibration performance of the HFLM model, calibration curves were plotted for all centers (Fig. 7). The results showed that the predicted probabilities were close to the actual outcomes, indicating good calibration of the model.

**Fig. 8:**
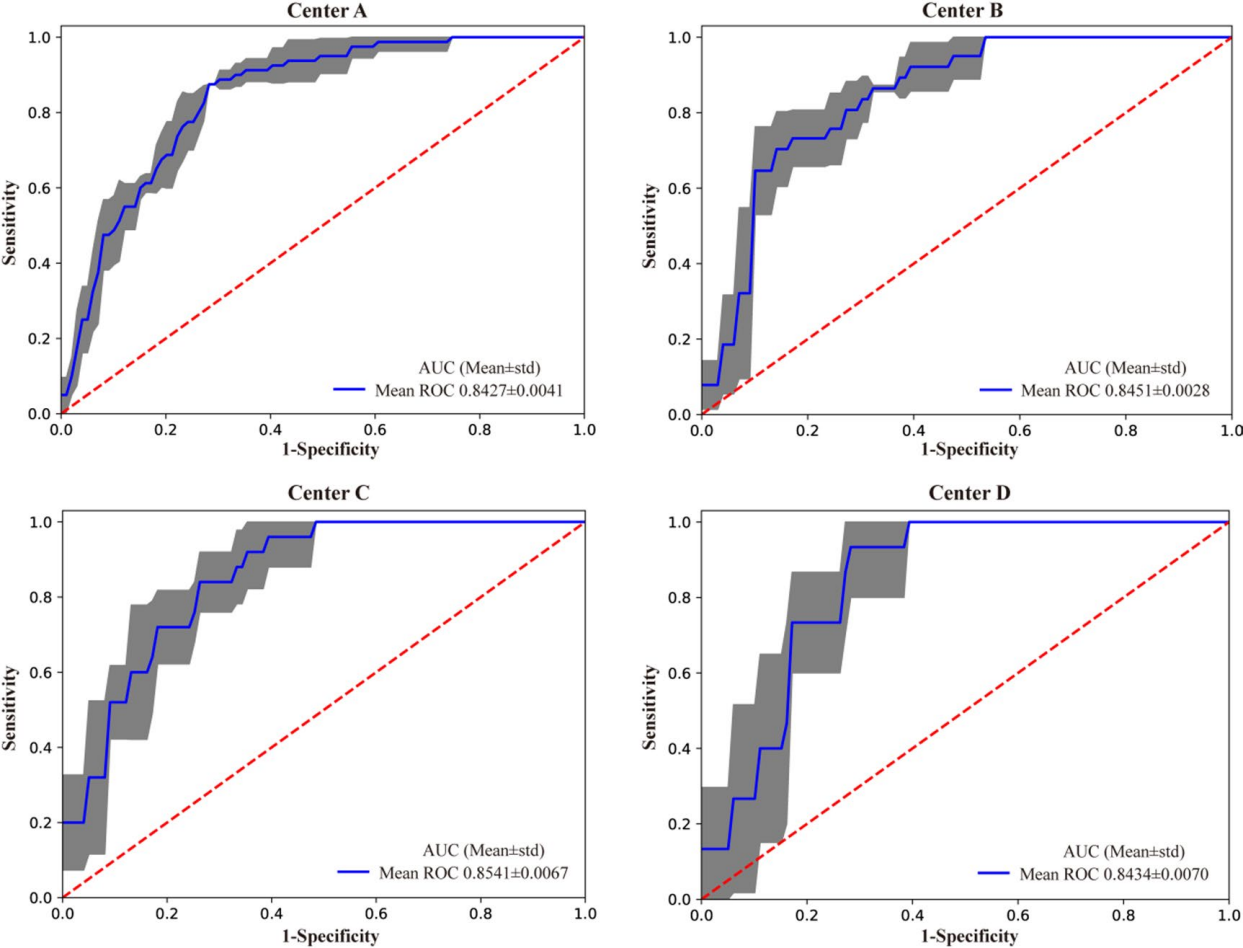
Five-fold cross-validation ROC curves for the four centers. The blue curve represents the average AUC of the fivefold curve. The grey areas represent the upper and lower limits of the ROC curve. The error band in the grey areas is the upper and lower boundary of the five-fold cross-verified ROC curve. Mean the AUC average for five-fold cross-validation, Std standard deviation.

In deep learning, model performance can be influenced by the distribution of the dataset. To further validate the generalization and stability of the model, we conducted five-fold cross-validation on the multi-center HFLM results. The average AUC values across the four data centers from the cross-validation were 0.8427 ± 0.0041, 0.8451 ± 0.0028, 0.8541 ± 0.0067, and 0.8434 ± 0.0070, respectively (Fig. 8). As shown in Fig. 8, each center had a high AUC (Area Under the ROC Curve), indicated that the model has strong generalization ability. The small standard deviations further indicate the model’s stability across different centers. For reference, the red dashed line in the ROC curves represents the random classification baseline.

**Fig. 9:**
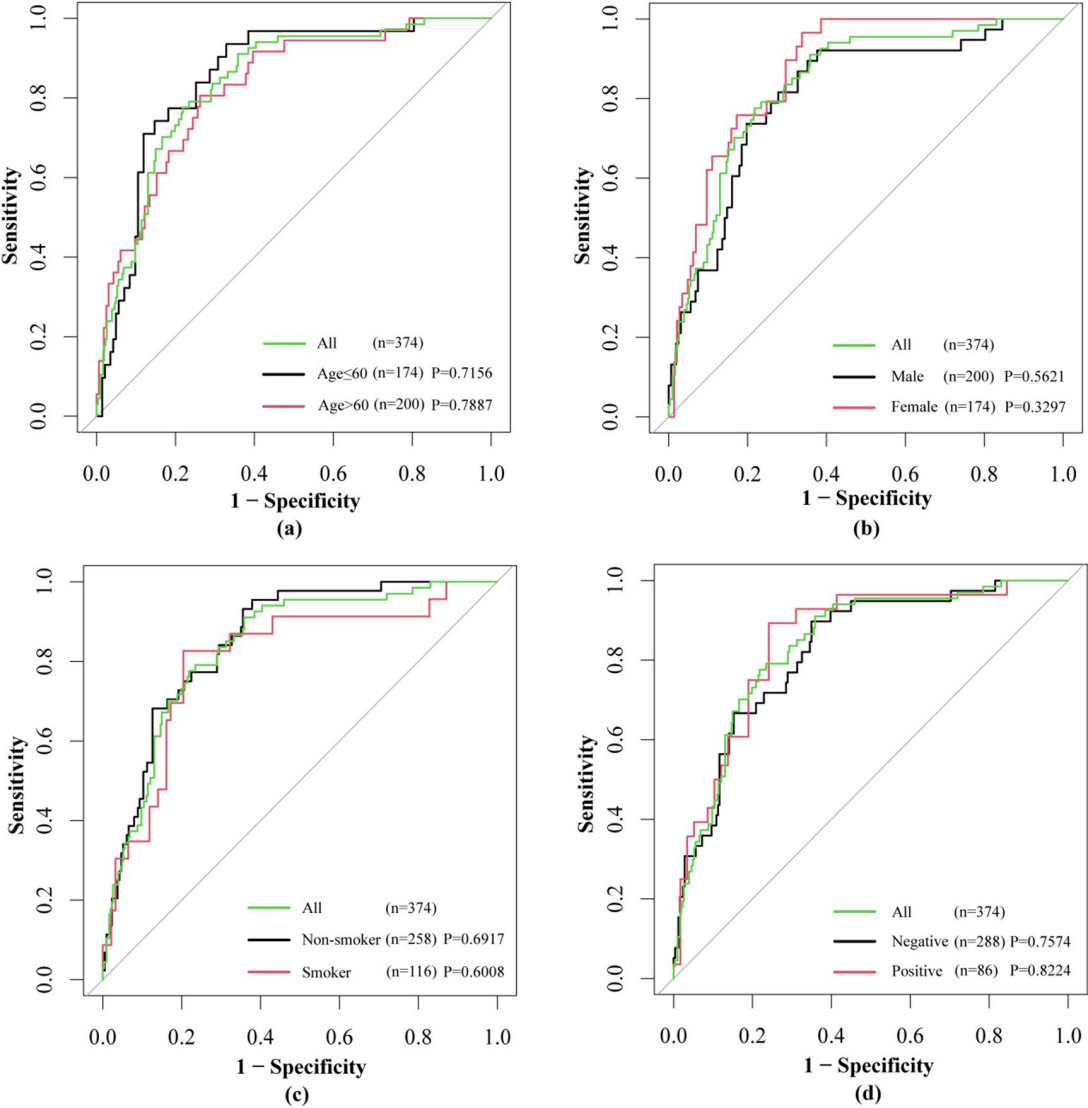
Stratified analysis results based on (**a**) age, (**b**) gender, (**c**) smoking history and (**d**) CEA. Statistical test: Delong test (two-tailed). P: significance value.

To evaluate whether the performance of the HFLM algorithm was independent of patient characteristics such as age, gender, smoking history, and CEA status, we conducted a stratified analysis. This analysis aimed to determine if the algorithm’s performance varied significantly based on these factors. The results demonstrated that the HFLM algorithm’s performance was not affected by patient age, gender, smoking history, or CEA status. The DeLong test confirmed that all p-values were greater than 0.05, indicating no statistically significant differences (Fig. 9).

**Fig. 10:**
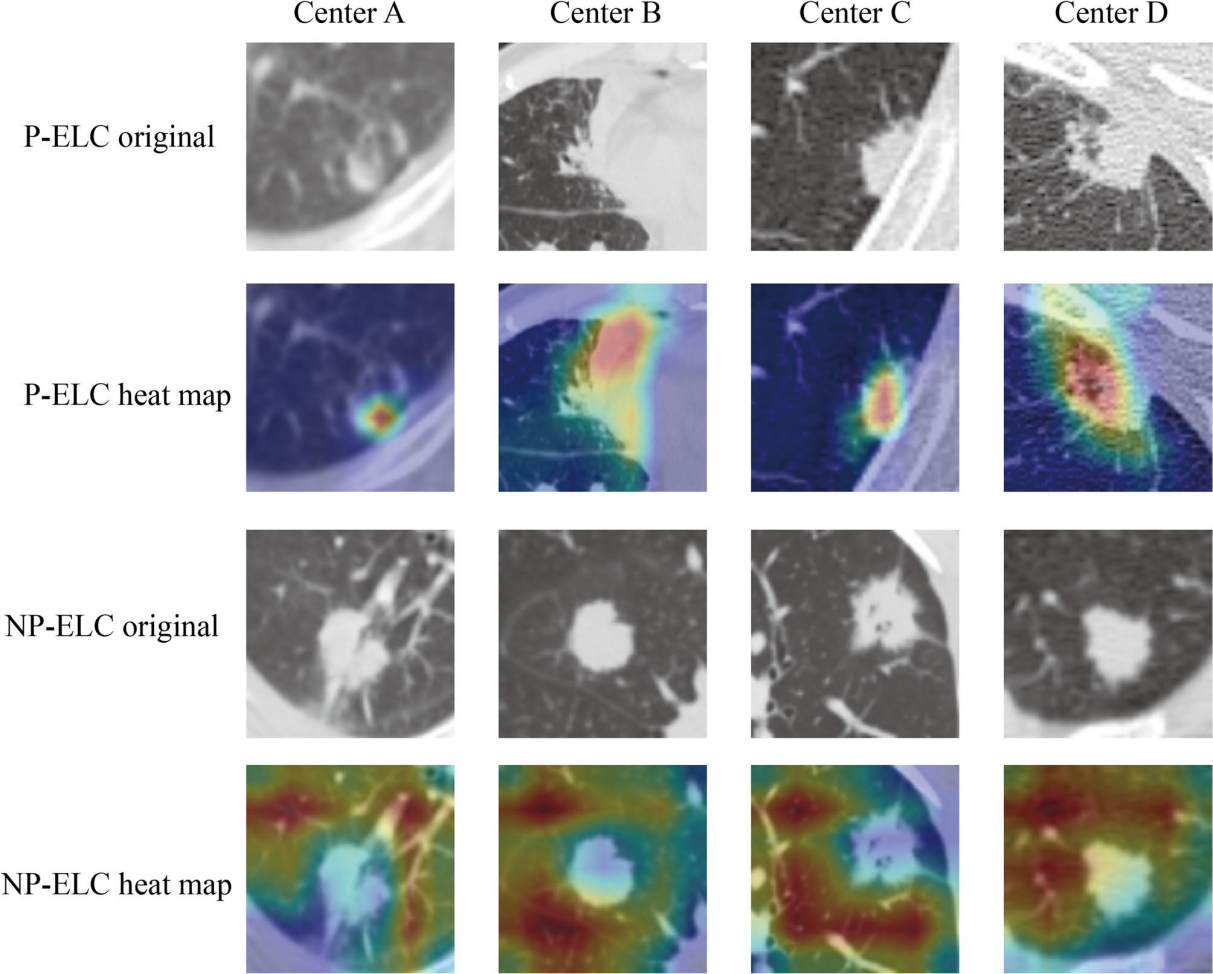
The heatmap shows the information acquired by the HFLM for images in the progressive and non-progressive classes.

### Interpretability visualization of HFLM

To investigate the interpretability and classification basis of HFLM in the two patient groups, we performed a class activation visualization analysis. As shown in Fig. 10, the visualization results of eight patients from four centers are presented, covering both non-progressive and progressive cases. The results indicate that the HFLM model mainly focuses on the tumor lesions and surrounding high-risk regions in progressive patients, showing more concentrated activation responses. In contrast, in non-progressive patients, the model’s attention regions are relatively scattered and primarily located in non-lesion tissue areas.

## Discussion

Currently, the TNM staging system is commonly used in early-stage lung cancer to assess prognosis by evaluating tumor size, lymph node involvement, and metastasis, providing a useful framework for treatment decisions^16^. However, the TNM system has significant limitations, as it does not account for tumor heterogeneity and individual patient characteristics like age and comorbidities, which can lead to varying outcomes despite similar stages. In contrast, deep learning models trained on CT images offer several advantages over the TNM system. These models can automatically extract detailed imaging features, integrate multi-modal data (such as genetic and clinical information), and provide more precise, personalized predictions.Deep learning models also reduce human error, improve sensitivity and accuracy, and are capable of continuous learning and adaptation through dynamic follow-up data, showing potential benefits in the prognosis assessment and treatment recommendations for lung cancer-specific survival rates^17,18^. As a result, deep learning models can provide a more comprehensive and accurate risk assessment for postoperative progression in early-stage lung cancer patients, assisting clinicians in making more informed decisions while reducing their clinical workload.

In recent years, deep learning has been used to predict the postoperative progression of NSCLC^19^. Coroller et al.^20^ used traditional deep learning features to predict the distant metastasis of lung cancer. In their study, they used radiomic features from CT images of NSCLC patients from a single center to predict pathological response. While this study provided valuable insights, it was conducted using data from a single medical center, which has certain limitations compared to multi-center studies. In this paper, we conducted research in a multi-center setting and proposed a generalized heterogeneous federated learning model (HFLM). This model aims to build personalized models with different architectures based on the specific conditions of different centers and reduces the impact of data heterogeneity on the global model. HFLM introduces a robust feature transfer strategy and defines a heterogeneous model interaction network. In this network, feature transfer is performed by calculating feature channel transfer weights and transfer quantities. Based on this, a feature transfer loss function is constructed to enhance the model’s focus on robust features during training, enabling effective feature identification and transfer, thus promoting information exchange between heterogeneous models.

Compared to clinical models, HFLM integrates CT imaging data from different centers through multi-center joint training, reduces the introduction of heterogeneous features through the robust feature transfer method, and enhances the generalization ability of the global model. At the same time, it maintains the personalized features of each center, providing a more accurate prediction of early-stage lung cancer prognosis. Experimental results also show that HFLM has better predictive performance.

Traditional federated learning algorithms typically require different centers to train models with the same structure^9–11^, without considering the specific conditions of each center. Current federated learning frameworks that accommodate heterogeneous models often either necessitate extra data^21,22^, risk reducing accuracy^23^, or by sharing prototypes through uploads to enable knowledge transfer across clients^15^. FedProto constructs class-level prototype representations by averaging the feature embeddings of local samples belonging to the same class and aggregates these prototypes on the server to achieve inter-center feature alignment. This prototype-based communication reduces parameter transmission and can partially mitigate data heterogeneity. In addition, KD-based federated learning frameworks adopt a teacher–student paradigm, in which the global model serves as the teacher, providing soft label outputs or feature representations to guide the learning of local models. This approach can improve convergence efficiency, but since the knowledge transfer is typically unidirectional, its adaptability to non-independent and identically distributed (non-IID) data is limited. These limitations significantly affect the versatility and practicality of federated learning. In contrast, HFLM allows each center to flexibly select and train different model architectures based on its specific conditions (such as data characteristics, computational resources, or technical capabilities), significantly lowering the barrier to participation and enhancing practicality. HFLM supports federated learning across different centers, and even when participating centers change, it can maintain stable operation without extensive retraining, improving the generality of federated learning. Experimental results further demonstrate that HFLM, by using the robust feature transfer strategy to reduce the introduction of heterogeneous features, achieved superior predictive performance in identifying patients with postoperative progression of early-stage lung cancer. Additionally, tests on generalization ability and stability further validate the robustness of HFLM.

Furthermore, as part of the federated learning framework, HFLM is designed to effectively protect patient data privacy. Specifically, the original imaging data at each center remain on local servers, and during local model training and global model aggregation, only model parameters are shared to enable feature knowledge transfer on the respective local data. This substantially reduces the risk of direct access to sensitive patient information. Nevertheless, potential privacy risks still exist, such as model inversion attacks, which could partially recover sensitive information from the shared parameters.

The present study had several limitations. Firstly, the study was retrospective, and therefore patient recruitment might be potentially biased. Secondly, the sample size used in the study was relatively small, and the class distribution across centers was imbalanced. For example, Center D had significantly fewer positive cases than negative cases. Such data characteristics may lead to insufficient learning of features for the minority class during model training, affecting the stability and reliability of predictions. We also observed that small sample size and class imbalance caused fluctuations in metrics such as AUC. Larger, more diverse datasets are needed to validate the findings and improve the model’s robustness.Third, during the process of deep learning feature extraction based on medical images, 3D images could provide deeper and more comprehensive features than 2D images.

## Conclusions

The study proposed a generalized heterogeneous federated learning model (HFLM) to address the challenges of model and data heterogeneity in multi-center settings for predicting postoperative progression in early-stage non-small cell lung cancer (NSCLC). The robust feature transfer strategy could enable effective collaboration across centers with varying model architectures while mitigating the impact of data heterogeneity.

The key findings demonstrated that HFLM achieved superior predictive performance, with AUC values ranging from 0.837 to 0.863 across four independent centers, outperforming both clinical models and traditional federated learning approaches (FedAvg, FedProx, Moon). The model’s robustness was further validated through cross-validation and stratified analyses, showing consistent performance regardless of patient demographics or data distribution variations.

This work contributes to existing knowledge by: (1) Introducing a flexible federated learning framework that accommodates heterogeneous model architectures, enhancing practicality for real-world clinical deployment. (2) Proving the efficacy of robust feature transfer in reducing the influence of data heterogeneity, thereby improving model generalizability.

In conclusion, HFLM successfully addresses critical barriers in multi-center federated learning, offering a scalable and accurate tool for postoperative risk assessment in early-stage NSCLC. The results align with the original research goal, supporting its potential to guide personalized treatment strategies while preserving data privacy.

## Supplementary Information

Below is the link to the electronic supplementary material.

Supplementary Material 1

## Data Availability

The datasets generated and/or analyzed during the current study are not publicly available because they contain sensitive information from a collaborating third-party institution. However, the data are available from the corresponding author upon reasonable request. Interested researchers may contact the corresponding author (181970902@qq.com) with a detailed research purpose, and data access will be granted under a signed data-sharing or non-disclosure agreement.

## References

[1] Siegel, R. L., Miller, K. D. & Jemal, A. Cancer statistics, 2018. *Cancer J. Clin.* **68** (1), 7–30 (2018). 10.3322/caac.21442 29313949

[2] Gridelli, C. et al. Non-small-cell lung cancer. *Nat. Reviews Disease Primers*. **1** (1), 1–16 (2015). 10.1038/nrdp.2015.9 27188576

[3] Uprety, D. et al. Neoadjuvant immunotherapy for NSCLC: current concepts and future approaches. *J. Thorac. Oncol.* **15** (8), 1281–1297 (2020). 32522713 10.1016/j.jtho.2020.05.020

[4] Woodard, G. A., Jones, K. D. & Jablons, D. M. Lung cancer staging and prognosis. *Lung Cancer Treat. Res.* **40**, 47–75 (2016). 10.1007/978-3-319-40389-2_3 27535389

[5] Wang, J. et al. Evaluation of the 7th edition of the TNM classification for lung cancer at a single institution. *J. Cancer Res. Clin. Oncol.* **140**, 1189–1195 (2014). 24676426 10.1007/s00432-014-1636-0 PMC11823730

[6] Shin, J. Y., Yoon, J. K. & Marwaha, G. Progress in the treatment and outcomes for early-stage non-small cell lung cancer. *Lung* **196**, 351–358 (2018). 29550987 10.1007/s00408-018-0110-1

[7] Heidari, A. et al. A new lung cancer detection method based on the chest CT images using federated learning and blockchain systems. *Artif. Intell. Med.* **141**, 102572 (2023). 37295902 10.1016/j.artmed.2023.102572

[8] Liu, Y. et al. Predicting treatment response in multicenter non-small cell lung cancer patients based on federated learning. *BMC Cancer*. **24** (1), 688 (2024). 38840081 10.1186/s12885-024-12456-7 PMC11155008

[9] McMahan, B. et al. Communication-efficient learning of deep networks from decentralized data. *Artif. Intell. Stat. PMLR* 1273–1282 (2017).

[10] Li, T. et al. Federated optimization in heterogeneous networks. *Proc. Mach. Learn. Syst.*** 2**, 429–450 (2020).

[11] Li, Q., He, B. & Song, D. Model-contrastive federated learning. In *Proceedings of the IEEE/CVF Conference on Computer Vision and Pattern Recognition*. 10713–10722. (2021).

[12] Jang, Y. et al. Learning what and where to transfer. In *International Conference on Machine Learning. PMLR*. 3030–3039 (2019).

[13] Toba, H. et al. Diagnosis of recurrence and follow-up using FDG-PET/CT for postoperative non-small-cell lung cancer patients. *Gen. Thorac. Cardiovasc. Surg.* **69**, 311–317 (2021). 32909168 10.1007/s11748-020-01477-1

[14] Shimada, Y. et al. Prognostic factors and the significance of treatment after recurrence in completely resected stage I non-small cell lung cancer. *Chest* **143** (6), 1626–1634 (2013). 23348916 10.1378/chest.12-1717

[15] Tan, Y. et al. Fedproto: Federated prototype learning across heterogeneous clients. In *Proceedings of the AAAI Conference on Artificial Intelligence*. Vol. 36(8). 8432–8440 (2022).

[16] Rami-Porta, R., Crowley, J. J. & Goldstraw, P. Review the revised TNM staging system for lung cancer. *Ann. Thorac. Cardiovasc. Surg.* **15** (1), 5 (2009). 19262443

[17] Avanzo, M. et al. Radiomics and deep learning in lung cancer. *Strahlenther. Onkol.* **196**, 879–887 (2020). 32367456 10.1007/s00066-020-01625-9

[18] She, Y. et al. Development and validation of a deep learning model for non–small cell lung cancer survival. *JAMA Netw. open.* **3** (6), e205842–e205842 (2020). 32492161 10.1001/jamanetworkopen.2020.5842 PMC7272121

[19] Kuang, Q. et al. Multimodal deep learning radiomics model for predicting postoperative progression in solid stage I non-small cell lung cancer. *Cancer Imaging*. **24** (1), 140 (2024). 39420411 10.1186/s40644-024-00783-8 PMC11487701

[20] Coroller, T. P. et al. Radiomic phenotype features predict pathological response in non-small cell lung cancer. *Radiother. Oncol.* **119** (3), 480–486 (2016). 27085484 10.1016/j.radonc.2016.04.004 PMC4930885

[21] Lin, T. et al. Ensemble distillation for robust model fusion in federated learning. *Adv. Neural. Inf. Process. Syst.* **33**, 2351–2363 (2020).

[22] Seo, H. et al. 16 federated knowledge distillation. *Mach. Learn. Wirel. Commun.* **2022**, 457 (2022).

[23] Shen, T. et al. Federated Mutual Learning. arxiv preprint arxiv:2006.16765 (2020).

