# Supplementary material for: A generalized heterogeneous federated model for identifying patients with postoperative progression of early-stage non-small cell lung cancer
Source: Sci Rep. 2025 Dec 1;16:910. doi: 10.1038/s41598-025-30565-6 (PMC12783761; doi:10.1038/s41598-025-30565-6)
Supplement: Supplementary file 1 — Supplementary Material 1 [file 41598_2025_30565_MOESM1_ESM.docx]

**Supplemental Materials**

**Supplementary S1: The heterogeneous federated learning model based on feature transfer.**

As shown in Figure S1, the algorithm in this paper consists of K+1 clients, each with its own local dataset. One of the clients is selected as the central server, which receives the local model parameters uploaded by the remaining K clients and then performs global model parameter aggregation and distributed. The schematic diagram of feature transfer is shown in Figure S2.

**Feature transfer:** There are *K* clients and one central server, denoted as *T_1_,T_2_,...,T_K_*和*S*, with the datasets of each center represented as {*D_1_...D_i_...D_K_，D_server_*}，*({x_i_},{y_i_})∈D_i_*. Among these, *{x_i_}* and *{y_i_}* refer to image data and image labels, respectively. The clients receive the global model parameters distributed by the central server and use both the global model parameters and the local model *i* parameters to extract features from the dataset *D_i_*. They then calculate the feature discrepancy based on equation (1)：

${{L_{2}=||r}_{\theta_{i}}(T_{\theta_{i}}^{n}(x))-S^{m}(x)||}_{2}^{2}$ $(1)$

In this equation, $T_{\theta_{i}}^{n}(x)$ represents the intermediate feature map from the *n* layer of local model *i*, while $S^{m}(x)$ denotes the intermediate feature map from the *m* layer of the global model. $r_{\theta_{i}}$ is a linear transformation parameterized by $\theta_{i}$, allowing pointwise convolution to ensure consistency in the number of feature maps between the two models.

**Local Model *i* Personalized Training:** When transferring features from the global model to train local model *i*, the process begins by defining a subnetwork *f* for the heterogeneous model interaction network *i*. This subnetwork takes the global model's features as input and outputs through a *softmax* layer. Subnetwork *f* computes the feature transfer weight $w_{c}^{m,n}$, which determines which features are selected for transfer to participate in the training of the local model. Next, a subnetwork *g* is defined for the heterogeneous model interaction network *i*. This subnetwork also takes the global model's features as input and outputs through a *ReLU6* activation layer. Subnetwork *g* then calculates the transfer amount $\lambda_{i}^{m,n}$ from the *m* layer of the global model to the *n* layer of the local model, $\lambda_{i}^{m,n}$>0. *f* and *g* together form the parameter $\phi_{i}$ of the heterogeneous model interaction network *i*, which is used for transferring features from source *S* to target *T_i_*. The transfer loss function is defined based on $L_{2}, w_{c}^{m,n}$ and $\lambda_{i}^{m,n}$ as follows:

$L_{wfm}^{m,n}(\theta_{i}|x,\phi_{i})=\sum_{(m,n\in\sigma)} \lambda_{i}^{m,n}\frac{1}{HW}\sum_{c} w_{c}^{m,n}\sum_{k,u} {({r_{\theta_{i}}(T_{\theta_{i}}^{n}(x))}_{c,k,u}-{S^{m}(x)}_{c,k,u})}^{2}$ $(2)$

Here, *H×W* represents the dimensions of the output feature maps from $r_{\theta_{i}}(T_{\theta_{i}}^{n}(x))$ and $S^{m}(x)$, *k*∈{1,2···,*H*}*,u*∈{1,2···,*W*}, $(m,n)\in\sigma$, and $\sigma$ refers to the predefined layer matching pairs between the models.

The training process of the local model based on feature transfer primarily includes the following steps: First, subnetworks *f* and *g* of model *i* compute $w_{c}^{m,n}$ and $\lambda_{i}^{m,n}$ based on the feature representations of the global model on the local dataset *D_i_*. Second, the loss function *L_wfm_* is constructed by incorporating the *L_2_* value to update the local model parameters $\theta_{i}$. In heterogeneous cases, according to equation (2), when there is a significant difference between the global and local model parameters, the representation difference on dataset *D_i_* increases, resulting in a larger *L_2_* value. This causes *L_wfm_* to place more emphasis on the features of the corresponding model layer. If these features fail to reduce the cross-entropy of the local model, the corresponding values of $w_{c}^{m,n}$ and $\lambda_{i}^{m,n}$ will decrease after updating the parameters of the low-heterogeneity feature selection network. Finally, the total loss function for training the local model *T_i_* parameters is:

$L_{todal}(\theta_{i}|x,y,\phi_{i},\theta_{global})=L_{org}(\theta_{i}|x,y)+\eta L_{wfm}(\theta_{i}|x,\phi_{i})$ $(3)$

In the equation, $L_{org}(\theta_{i}|x,y)$ represents the cross-entropy loss of *T_i_*, and $\eta$>0 is a hyperparameter. The optimization objective of the local model *T_i_* is:

${min}_{\theta_{i}}E_{(x,y)\sim D_{i}}(L_{todal}(\theta_{i}|x,y,\phi_{i},\theta_{global}))$ $(4)$

**Global Model Aggregation:** When aggregating global model parameters, to address the issue of model heterogeneity and enhance the model's focus on robust features, it is proposed to transfer features from each local model. A dynamic aggregation network is used to guide the feature transfer from local models, allowing the global model to aggregate parameters during the transfer process. Therefore, the optimization loss function of the global model is:

$L_{global}(\theta_{global}|x,\varepsilon)=L_{org}(\theta_{global}|x,y)+\eta L_{wfm}(\theta_{global}|x,\varepsilon)$ $(5)$

In the equation, $\varepsilon$ represents the parameters of the dynamic aggregation network, which consists of *N* sets of heterogeneous model interaction networks. The global model aggregation optimization term is:

${min}_{\theta_{global}}\sum_{Q=1}^{N} E_{(x,y)\sim D_{global}}(L_{todal}(\theta_{global}|x,\varepsilon))$ $(6)$

Here, *Q* represents the feature transfer from *T_i_* to the *server*, and $\varepsilon$ denotes the parameters of the dynamic aggregation network *i*. When the server receives the parameters from each local model, it first computes $w_{c}^{m,n}$ and $\lambda_{Q}^{m,n}$ based on the representation of *T_i_* on *D_global_*. Then, it updates the server parameters using equation (5), performing this update a total of *N* times. During the aggregation of the global model parameters according to equation (6), the representations of *D_global_* differ due to variations in the parameters of different local models. While $\varepsilon$ provides $w_{c}^{m,n}$ and $\lambda_{Q}^{m,n}$ only for the representations during *Q* feature transfers, it can enhance $w_{c}^{m,n}$ and $\lambda_{Q}^{m,n}$ for features that effectively reduce the server's cross-entropy. Therefore, only features that remain consistently effective throughout the *N* transfers will be assigned higher values of $w_{c}^{m,n}$ and $\lambda_{Q}^{m,n}$. Additionally, during the aggregation process, the global model will pay more attention to these features.

In each cycle of information exchange, the *server* must both receive the local model parameters from various clients and transmit the global model parameters to each client. Both *T_i_* and *S* update their model weight parameters using the stochastic gradient descent method, along with the parameters of the heterogeneous model interaction network and the dynamic aggregation network.

**Supplementary S2: Deep feature extraction and construction of the Sparse Bayesian Extreme Learning Machine**

**Deep feature extraction:** To fully leverage the features within the convolutional neural network (*CNN*), this study extracts features from local datasets using network models trained at different centers. Features maps generated by all filters across the convolutional layers are read, and the mean of each feature map is computed to obtain the corresponding filter's deep features. These deep features are then concatenated horizontally to produce the overall deep feature representation of the image across the entire network.

To enhance model prediction performance and reduce computational complexity, it is essential to select features highly relevant to the task. In this study, the Mann-Whitney U test, a statistical method, is applied to filter the extracted deep features. Following this, the Maximum Relevance Minimum Redundancy (*mRMR*) algorithm is used to perform dimensionality reduction on the features selected by the U test, thereby improving the relevance between features and the task while eliminating redundancy among features. Finally, the filtered deep features are used for classification.

**The Sparse Bayesian Extreme Learning Machine:** Extreme Learning Machine (*ELM*) is a novel single-hidden-layer feedforward neural network algorithm. Its main feature is the random assignment of the connection weights *w* between the input layer and the hidden layer, along with the biases *b*. *ELM* only requires setting the network structure to determine the corresponding output matrix, boasting faster learning speeds and better generalization capabilities. This paper aims to construct a classifier based on a Sparse Bayesian Extreme Learning Machine, combining the rapid learning capability of *ELM* with the effectiveness of sparse Bayesian methods. Additionally, by introducing the *L_1_* norm into the optimization process of *ELM*, the model is constrained to yield sparse solutions, thereby optimizing the model's complexity and generalization ability.

**Supplementary S3: Construction of clinical models**

In this study, to mitigate multicollinearity among features, clinical characteristics were evaluated using the Variance Inflation Factor (VIF) method. Ultimately, four clinical indicators of Age, Gender and Smoking history and CEA status were selected to construct clinical models based on data from the four centers using a multivariate logistic regression algorithm

**Supplementary S4 Calculation of confidence intervals**

The AUC and its 95% confidence interval (CI) of the ROC curve were calculated using DeLong’s test as follows:

1. ROC curve and AUC calculation: First, the ROC curve was constructed based on the predicted probabilities and true labels, and the corresponding AUC was calculated.
2. DeLong’s test: The standard error (SE) of the AUC was estimated using DeLong’s method, which is a nonparametric approach that accounts for the correlation between positive and negative samples within the same dataset.

3. Confidence interval calculation: Based on the standard error of the AUC, the 95% CI was computed using the normal approximation as follows:

CI_95%_=AUC±Z_0.975_×SE_AUC_

where Z_0.975_≈1.96 is the 97.5th percentile of the standard normal distribution.


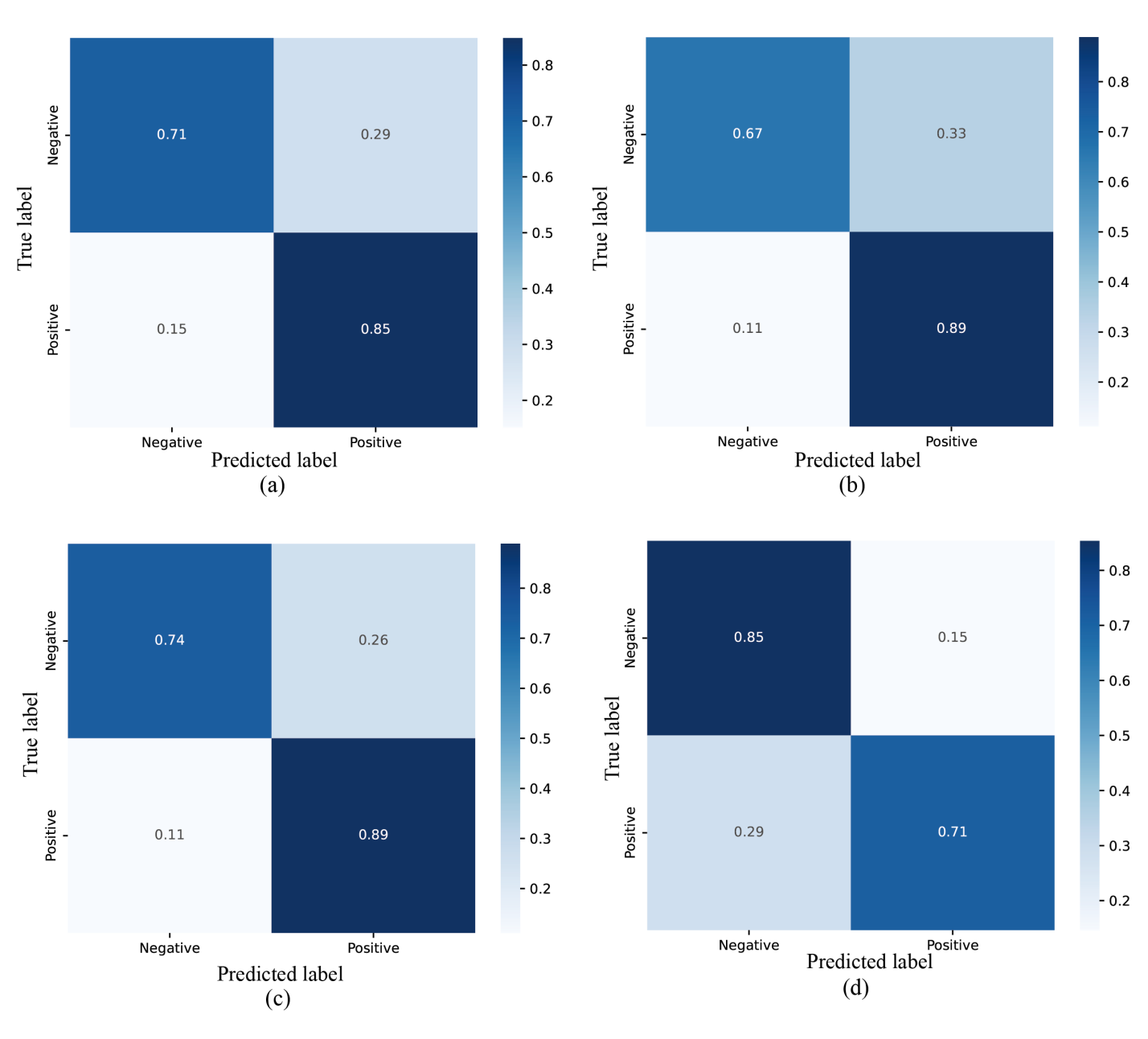
**Figure S1 The confusion matrices for each center.**

| **Table S1 CT Scanners and technique parameters used for chest CT examination in four Medical Centers** | | | | | | | | |
| --- | --- | --- | --- | --- | --- | --- | --- | --- |
| **Center** | **CT Scanner** | **Company** | **Tube voltage**  **(kV)** | **Pitch** | **Contrast Agent** | **Reconstruction kernel** | **Slice thickness (mm)** | **Image Matrix** |
| A | Siemens Force Definition | Siemens Medical Solutions, Forchheim, Germany | 120 | 1.20 | Iohexol  (350 mgI/mL)  Iodixanol  (350 mgI/mL) | B30f | 0.75 | 512 X 512 |
|  | Toshiba Aquilion 64 Slice CT Scanner | Toshiba Medical Systems, Tokyo, Japan | 120 | 1.35 | Iohexol  (350 mgI/mL)  Iodixanol  (350 mgI/mL) | FC03 | 1.25 | 512 X 512 |
|  | GE Discovery CT750 HD | GE Healthcare, Milwaukee, Wisconsin | 120 | 1.375 | Iohexol  (350 mgI/mL)  Iodixanol  (350 mgI/mL) | Standard | 0.625 | 512 X 512 |
| B | GE LightSpeed Ultra | GE Healthcare, Milwaukee, Wisconsin | 120 | 1.50 | Iohexol  (350 mgI/mL)  Iodixanol  (350 mgI/mL) | Standard | 1.25 | 512 X 512 |
|  | Philips Brilliance iCT | Koninklijke Philips N.V, Amsterdam, the Netherlands | 120 | 1.20 | Iohexol  (350 mgI/mL)  Iodixanol  (350 mgI/mL) | C | 0.625 | 512 X 512 |
| C | Siemens Force Definition | Siemens Medical Solutions, Forchheim, Germany | 120 | 1.20 | Iohexol  (350 mgI/mL)  Iodixanol  (350 mgI/mL) | B30f | 0.75 | 512 X 512 |
|  | Philips Brilliance iCT | Koninklijke Philips N.V, Amsterdam, the Netherlands | 120 | 1.20 | Iohexol  (350 mgI/mL)  Iodixanol  (350 mgI/mL) | C | 0.625 | 512 X 512 |
| D | Siemens Force Definition | Siemens Medical Solutions, Forchheim, Germany | 120 | 1.20 | Iohexol  (350 mgI/mL)  Iodixanol  (350 mgI/mL) | B30f | 0.75 | 512 X 512 |
|  | UIH uCT 760 | United Imaging Healthcare, Shanghai, China | 120 | 1.20 | Iohexol  (350 mgI/mL)  Iodixanol  (350 mgI/mL) | Soft | 0.625 | 512 X 512 |

| **Table S2 The pseudocode of the HFLM** | | |
| --- | --- | --- |
| ***Initialization Phase*** | ***Aggregation Phase*** | ***Local Model Training Phase*** |
| *Input: Information exchange rounds* ***T****,* *model training epochs* ***E****, number of inner feature transfer iterations* ***G****, number of clients* ***N****, learning rate* ***α****, data* ***D_global_*** *_..._* ***D_K_*** | *for t = 1 to T do* | $\text{θ}_{\text{global}}^{\text{t}\text{+1}}$*=*$\text{θ}_{\text{global}}^{\text{t}\text{,N}}$  *//Send global model parameters to Client k* |
| *Output：Global model* $\text{θ}_{\text{global}}^{\text{T}}$ *, Local models* $\text{θ}_{\text{1}}^{\text{T}}$*，*$\text{θ}_{\text{2}}^{\text{T}}$*，...，*$\text{θ}_{\text{N}}^{\text{T}}$ | *Global model aggregation*  *(t，{ θ_k_ }):*$\text{θ}_{\text{global}}^{\text{t,k}}$*=*$\text{θ}_{\text{global}}^{\text{t}}$ | *for k = 1 to N do* |
| *Initialize model parameters:* $\text{θ}_{\text{global}}^{\text{1}}$*，*$\text{θ}_{\text{1}}^{\text{1}}$*，*$\text{θ}_{\text{2}}^{\text{1}}\text{，}$*...，*$\text{θ}_{\text{N}}^{\text{1}}$ | *for k = 1 to N do* $\text{θ}_{\text{global}}^{\text{t,k,j}}$*=*$\text{θ}_{\text{global}}^{\text{t,k}}$ | *Local model training (t,k,θ_global_):*$\text{θ}_{\text{k}}^{\text{t}\text{,j}}$*=*$\text{θ}_{\text{k}}^{\text{t}}$ |
| *Feature transfer: (*$\text{θ}_{\text{k}}^{\text{j}}\text{,}\text{ϕ}_{\text{k}}^{\text{j}}$*):* $\text{θ}_{\text{k}}^{\text{j,g}}$*=*$\text{θ}_{\text{k}}^{\text{j}}$ | *for j = 1 to E do* | *for j = 1 to E do；* |
| *for g = 1 to G do* | *Feature ← Clientθ_k_ (D_global_ )*  *//Extract data features using local model parameters* | *Feature ← Serverθ_global_ (D_k_ )*  *//Extract data features using global model parameters* |
| $\text{θ}_{\text{k}}^{\text{j,g+1}}$*←* $\text{θ}_{\text{k}}^{\text{j,g}}$*- α*$\text{∇}_{\text{θ}}\text{L}_{\text{wfm}}\text{(}\text{θ}_{\text{k}}^{\text{j,g}}\text{\vert x,}\text{ϕ}_{\text{k}}^{\text{j}}\text{)}$  *//Compute loss using the features extracted by the two models* | $\text{θ}_{\text{global}}^{\text{t,k,j+1}}$*，*$\text{ε}$*^j+1^ ← feature transfer (*$\text{θ}_{\text{global}}^{\text{t,k,j}}$*,*$\text{ε}$*^j^)* | $\text{θ}_{\text{k}}^{\text{t}\text{,j+1}}$*，*$\text{ϕ}_{\text{k}}^{\text{j+1}}$ *← feature transfer (*$\text{θ}_{\text{k}}^{\text{t,j}}$*,*$\text{ϕ}_{\text{k}}^{\text{j}}$*)* |
| $\text{θ}_{\text{k}}^{\text{j+1}}$*←* $\text{θ}_{\text{k}}^{\text{j,G}}$*- α*$\text{∇}_{\text{θ}}\text{L}_{\text{org}}\text{(}\text{θ}_{\text{k}}^{\text{j,G}}\text{\vert x,y}\text{)}$ | $\text{θ}_{\text{global}}^{\text{t}\text{,}\text{k+1}}$*=*$\text{θ}_{\text{global}}^{\text{t}\text{,}\text{k,E}}$ | $\text{θ}_{\text{k}}^{\text{t+1}}$*=*$\text{θ}_{\text{k}}^{\text{t,E}}$  *//Send local model parameters to the Server* |
| $\text{ϕ}_{\text{k}}^{\text{j+1}}$*←* $\text{ϕ}_{\text{k}}^{\text{j}}$*- α*$\text{∇}_{\text{ϕ}}\text{L}_{\text{org}}\text{(}\text{θ}_{\text{k}}^{\text{j+1}}\text{\vert x,y}\text{)}$ |  | *...* |
| *return* $\text{θ}_{\text{k}}^{\text{j+1}}$*,*$\text{ϕ}_{\text{k}}^{\text{j+1}}$ |  | *return* $\text{θ}_{\text{global}}^{\text{T}}$*，*$\text{θ}_{\text{1}}^{\text{T}}$*，*$\text{θ}_{\text{2}}^{\text{T}}\text{，}$*...，*$\text{θ}_{\text{N}}^{\text{T}}$ |

| **Table S3 Experimental parameter** | | | |
| --- | --- | --- | --- |
| learning rate | *0.01* | batch size | *32* |
| optimizer | *SGD* | alpha | *0.05* |
| momentum | *0.9* | weight decay | *0.0001* |
| image input shape | *3×224×224* | seed | *4000* |
| loss function | *focalloss* | local model | *VGG16* |
| global model | *ResNet18* |  |  |

| Table S4 Performance tables of four central data test sets for clinical models and HFLM | | | | | |
| --- | --- | --- | --- | --- | --- |
| Method | Evaluation | CenterA | CenterB | CenterC | CenterD |
| Clinical  model | AUC | 0.490(0.4070-0.6175) | 0.674(0.5265-0.8186) | 0.469(0.2437-0.6884) | 0.394(0.4047-0.8183) |
|  | Sensitive | 0.2727(9/33) | 0.6667(12/18) | 0.3333(3/9) | 0.0000(0/7) |
|  | Specificity | 0.5685(83/146) | 0.6818(45/66) | 0.7222(39/54) | 0.7317(30/41) |
|  | Accuracy | 0.5140(92/179) | 0.6786(57/84) | 0.6667(42/63) | 0.6250(30/48) |
|  | PPV | 0.1250(9/72) | 0.3636(12/33) | 0.1667(3/18) | 0.0000(0/11) |
|  | NPV | 0.7757(83/107) | 0.8824(45/51) | 0.8667(39/45) | 0.8108(30/37) |
| HFLM | AUC | 0.863(0.8072-0.9192) | 0.837(0.7204-0.9530) | 0.846(0.7349-0.9564) | 0.847(0.6971-0.9963) |
|  | Sensitive | 0.8485(28/33) | 0.8889(16/18) | 0.8889(8/9) | 0.7143(5/7) |
|  | Specificity | 0.7123(104/146) | 0.6667(44/66) | 0.7407(40/54) | 0.8537(35/41) |
|  | Accuracy | 0.7374(132/179) | 0.7143(60/84) | 0.7619(48/63) | 0.8333(40/48) |
|  | PPV | 0.4000(28/70) | 0.4211(16/38) | 0.3636(8/22) | 0.4545(5/11) |
|  | NPV | 0.9541(104/109) | 0.9565(44/46) | 0.9756(40/41) | 0.9459(35/37) |
| HFLM: heterogeneous federated learning model, AUC: area under the curve, PPV: positive predictive value, NPV: negative predictive value. | | | | | |
